# Genetic diversity and relationships of broomcorn millet based on *trn*T*-trn*L and *GBSSI* sequences

**DOI:** 10.1371/journal.pone.0325433

**Published:** 2025-07-23

**Authors:** Xiaohan Yu, Funa Tan, Xiaoxing Wang, Jiandong Ren, Shaoxiong Liu, Yue Wang, Xuxia Xin, Ruonan Wang, Yingxing Zhang, Zhaoyan Chen, Jishan Xiang, Minxuan Liu

**Affiliations:** 1 Institute of Crop Sciences, State Key Laboratory of Crop Gene Resources and Breeding, Chinese Academy of Agricultural Sciences, Beijing, China; 2 College of Biological Sciences and Technology, YiLi Normal University, Yili, China; 3 Chifeng University, Chifeng, China; Ben-Gurion University, ISRAEL

## Abstract

Broomcorn millet (*Panicum miliaceum* L.) is the oldest crop originating in China. The routes of transmission have been the focus of broomcorn millet research. This study evaluated genetic diversity and relationship of 430 broomcorn millet accessions (369 domestic accessions from nine regions and 61 foreign accessions from twenty-four counties) based on the chloroplast DNA *trn*T-*trn*L spacer sequence and nuclear DNA *GBSSI* sequence to explore the domestication of broomcorn millet. The *trn*T-*trn*L sequence was highly conserved, while the diversity of *GBSSI* sequence was significantly higher. Results of this study suggest that broomcorn millet may have originated from the core area (including Shanxi, Shaanxi, Inner Mongolia, Ningxia and Gansu) and then spread westward to Xinjiang and into Eurasia, or eastward from Shanxi to Hebei, Inner Mongolia and northeast China. Xinjiang is crucial for broomcorn millet to spread westward. This study revealed the genetic diversity of broomcorn millet accessions from different geographical sources, laying a theoretical foundation for further analysis of the evolutionary origin of this taxon.

## 1 Introduction

Broomcorn millet (*Panicum miliaceum* L.) was originated from China and domesticated approximately 10,000 years ago [1,2], mainly cultivated in semi-arid regions in Asia and Europe [3]. It has short growth cycles and low water and nutrient requirements, as well as high salt and alkali resistance, making it an excellent crop for overcoming abiotic stresses [4,5]. In addition, broomcorn millet is rich in protein, vitamins, calcium, iron, zinc, and other mineral elements. Therefore, it is also a crop with high commercial value. The total cultivation area of broomcorn millet has been maintained at about 1.5 million ha in recent decades.

To develop new germplasm resources, the genetic diversity and relationships of broomcorn millet populations need to be compared and evaluated. At present, research on that mainly involves morphological and molecular markers, such as SSRs, AFLPs, and RAPD markers [6–8]. Although DNA sequencing technology is commonly used to explain the diversity and reveal the evolutionary processes in many crops due to its advantages of high accuracy, the application of DNA sequences to study broomcorn millet is relatively rare. Common nuclear genome sequences used in analysis include *ITS*, *GBSSI*, and *FLO/LFY* [4,9]. Li *et al.*[10] used ITS and ETS sequences to conduct molecular identification of broomcorn millet remains dug from Xiaohe cemetery, which provided key information about the domestication and spread of broomcorn millet. The chloroplast genome is single-parent, with small molecular weight, simple structure and relatively conservative sequence, making it a good choice for studying the relationship between crop species. At present, commonly used chloroplast genome sequences include *rbc*L, *ndh*F, *mat*K and *trn*T-*trn*L spacer sequences [10]. Hollingsworth *et al.* [11] and Aliscioni *et al.* [12] used *rbc*L, *mat*K, and *ndh*F to study broomcorn millet species and found that the *rbc*L and *mat*K markers could identify broomcorn millet species, but they couldn’t effectively distinguish phylogeny; the *ndh*F gene failed to differentiate species. Meng [13] analyzed the genetic diversity of broomcorn millet, explored its relationship with some wild relatives based on the non-coding region sequence of chloroplast DNA, he found that the cpDNA non-coding region sequence was conserved within broomcorn species, but there was polymorphism among species. In the phylogenetic tree based on the *trn*H-*psb*A sequence, broomcorn millet and *Pamicum capillare* were clustered together, and Meng [13] speculated that *Pamicum capillare* might be the ancestor of broomcorn millet.

In this study, a large number of broomcorn millet accessions from thirty-three countries or regions were used. By analyzing the sequence polymorphisms and phylogenetic trees of 430 broomcorn millet accessions based on the cpDNA *trn*T-*trn*L and nrDNA *GBSSI* sequences, we obtained more genetic information of broomcorn millet accessions, and the results supported the origination of broomcorn millet in China. The resulting data will lay the foundation for the origin and evolution of broomcorn millet.

## 2 Materials and methods

### 2.1 Plant materials

A set of 430 broomcorn millet accessions from the Institute of Crop Sciences of the Chinese Academy of Agricultural Sciences were used (Table 1). The samples included 369 domestic accessions and 61 foreign accessions. These accessions were divided into 15 populations according to their source, including Ningxia, Shanxi, Inner Mongolia, Hebei, Liaoning, Heilongjiang, Shaanxi, Xinjiang, and Gansu population of China and Asia, Europe, South America, North America, Oceania, and Africa population respectively. Additional information about broomcorn millet accessions was given in S1 Table.

**Table 1:** Region and number of materials from each country of broomcorn millet.

Number	Population	Continent	Region of materials	Number of materials
**1**	Ningxia	**Asia**	Ningxia, China	23
2	Shanxi		Shanxi, China	14
3	Inner Mongolia		Inner Mongolia, China	22
4	Hebei		Hebei, China	35
5	Liaoning		Liaoning, China	13
6	Heilongjiang		Heilongjiang, China	32
7	Shaanxi		Shaanxi, China	14
8	Xinjiang		Xinjiang, China	77
9	Taiwan		Gansu, China	139
10	Other Asian countries		Taiwan, China	1
			Georgia	2
			Afghanistan	9
			India	2
			Pakistan	4
			Turkey	1
			Nepal	5
			Kazakhstan	1
11	Europe	**Europe**	France	1
			Germany	3
			Hungary	4
			England	1
			Ukraine	3
			Soviet Union	4
			Belgium	1
			Romania	1
			Bulgaria	1
			Czechoslovakia	2
12	South America	**South America**	Argentina	2
13	North America	**North America**	Canada	1
			America	4
14	Oceania	**Oceania**	Australia	5
15	Africa	**Africa**	Morocco	2
			Kenya	1
		**Total**		430

### 2.2 DNA extraction

Genomic DNA was isolated by the DNA extraction kit provided by Beijing Dingguo Changsheng Biological Co. Ltd using 50 mg young leaf tissue. The quality of the DNA was checked on 1% agarose gel electrophoresis, and the DNA concentration was checked on an ultra-micro spectrophotometer (P360). The final concentration of each DNA sample was adjusted to 30 ng·μL ^−1^ and stored at −20°C.

### 2.3 Primer design and synthesis

The cpDNA *trn*T-*trn*L sequence with ID JQ972982.1 and nrDNA *GBSSI* sequence with ID FJ430153.1 of broomcorn millet were retrieved from GenBank. Primers were designed using Primer 5.0 software. The primers used for *trn*T-*trn*L were forward 5’-ATGCGATGCTCTAACCTCTG-3’ and reverse 5’-CAATCAAGTCCGTAGCGTCT-3’. The primers for *GBSSI* were forward 5’-CACCGTGAGCCCCTACTACGCCGAG-3’ and reverse 5’- TACCGTGCCGTATCGCATCCCCT-3’. All primers were synthesized by Shanghai Yingjie Jieji Trading Co. Ltd.

### 2.4 PCR amplification and sequencing

PCR amplification reactions were carried out in a MY-CYCLER PCR. Polymerase chain reaction (PCR) of *trn*T-*trn*L sequence was conducted with a reaction volume of 50 μL, containing 5 μL 10 × PCR buffer, 4 μL 2.5 mmol·L^-1^ dNTP, 0.5 μLTaq polymerase (5.0 U·μL ^−1^), 1 μL primers, 2 μL DNA, and 36.5 μL ddH_2_O. The following PCR program was used: initial denaturation of 5 min at 95^o^C, followed by 35 cycles of denaturation at 30 s at 95^o^C, annealing for 30 s at 57^o^C, extension for 1 min at 72^o^C, and a final extension for 10 min at 72^o^C.

Polymerase chain reaction (PCR) amplifications of *GBSSI* were performed in a total volume of 50 μL, containing 2 μL DNA, 2 μL primers, 44 μL 1 × T3 Super PCR Mix. The following PCR program was used: initial denaturation of 2 min at 98^o^C, followed by 30 cycles of denaturation at 10 s at 98^o^C, annealing 10 s at 57^o^C, extension for 1 min at 72^o^C, and a final extension for 5 min at 72^o^C. The amplified products were visualized on 1% agarose gel electrophoresis and sequenced by Shanghai Bioengineering Co. Ltd.

### 2.5 Data analysis

The sequences were spliced using DNAMAN software. MEGA7.0.26 was used for multiple sequence alignment and calculating the following measures of the sequence: sequence length, conserved sites, variable sites, and parsimony informative sites. Genetic distance between populations and within populations was evaluated by MEGA7.0. A phylogenetic tree was estimated using Jukes-Cantor distances with the Neighbor-Joining method (N-J). DnaSP5.0 software was used to analyze sequence diversity including nucleotide diversity (π) and segregating site polymorphisms (θ_w_). Gene flow (Nm), the coefficient of genetic differentiation (Fst), Tajima’s D value, Fu and Li’s D*, and Fu and Li’s F* were calculated using DnaSP5.0.

## 3 Results

### 3.1 Analysis of *trnT-trnL* and *GBSS*I sequence features

The aligned *trn*T*-trn*L sequence yielded a total of 761 sites, including 733 conserved sites, 10 variable sites, two parsimony informative sites, eight singleton variable sites, 18 indel sites, and the G + C content was 25.4%. The accession from Gansu, China had the most abundant variation sites (10 variable sites including 1 parsimony informative site). There were no variable sites and indels in accessions from Shaanxi, Europe, South America, North America, Oceania, and Africa. Length variation and base composition of aligned *GBSSI* sequences from the studied accessions were 690 bp long, including 194 conserved sites, 420 variable sites, 376 parsimony informative sites, 44 singleton variable sites, 76 indel sites, and a G + C content of 59.5%. The analysis of the *GBSSI* sequences showed that the accessions from Gansu, China had the most abundant variable sites, with a total of 700 sites, including 351 variable sites and 62 indels. The G + C content and the number of variable sites in *GBSSI* sequence were significantly higher than those in *trnT-trnL* sequence, indicating that more variation is contained in GBSSI sequence. In analysis of both *trnT-trnL* and *GBSSI* sequences, the most abundant variable sites were found in Gansu accession.

### 3.2 Analysis of genetic diversity of *trnT-trnL* and *GBSSI* sequences of broomcorn millet

**Fig 1:**
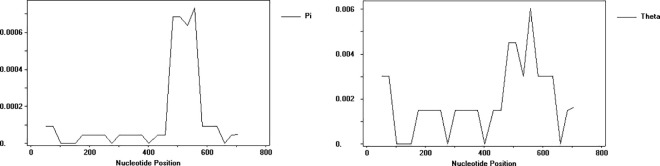
The variations of nucleotide diversity (π) and segregating site polymorphisms (θ_w_) in *trn*T-*trn*L.

**Fig 2:**
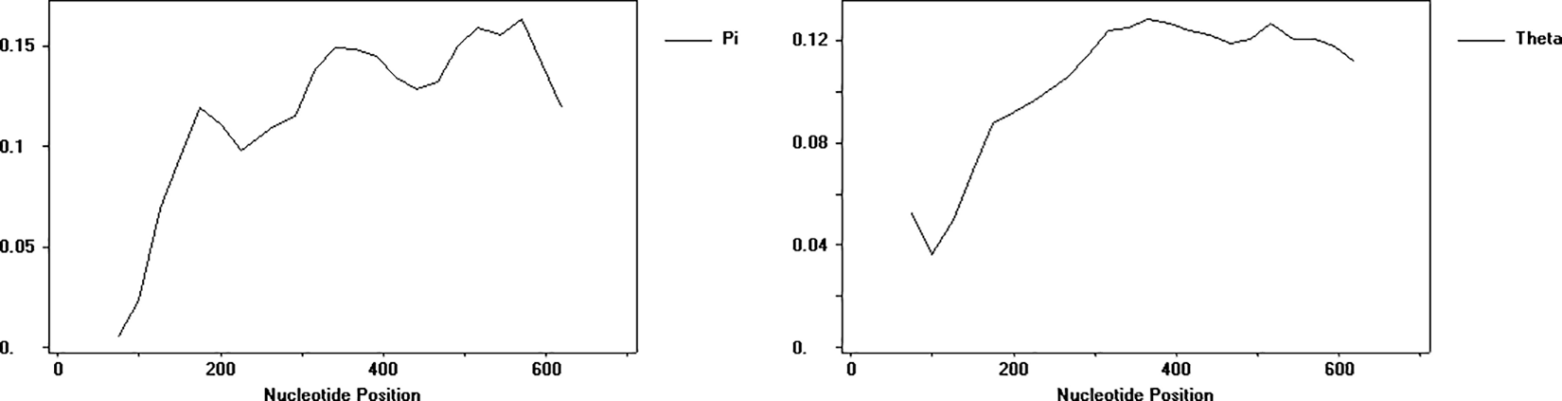
The variations of nucleotide diversity (π) and segregating site polymorphisms (θ_w_) in *GBSSI.*

The total number of segregating sites (S) of 430 accessions was 10, the haplotype diversity (Hd) was 0.0640, the nucleotide diversity (π) was 0.00014, and the average number of segregating site polymorphisms (θ_w_) was 0.00203 for the *trn*T-*trn*L sequence. Fig 1 shows the trend of variations of nucleotide diversity (π) and segregating site polymorphisms in *trn*T-*trn*L, and the highly variable sites were concentrated in the 450–600 bp region. The *GBSSI* sequence is rich in polymorphisms. It had 420 segregating sites (S) and the haplotype diversity (Hd) was 0.9976. The nucleotide diversity (π) was 0.11025, and the average number of segregating site polymorphisms (θ_w_) was 0.13221. There were some differences in the changes of π and θ_w_ in different regions (Fig 2), indicating that the distribution of the sequence polymorphisms in broomcorn millet *GBSSI* is uneven in each region.

As evidenced by the estimates of wild accessions and local varieties polymorphisms of the *trn*T-*trn*L sequence listed in Table 2, the Hd value of local varieties (0.068) was higher than that of wild resources (0.046), which might be due to the abundant source of local varieties. The Hd value of Inner Mongolian wild population in China was the highest at 0.182; the wild accession from Ningxia, China was second at 0.133. The π and θ_w_ values of the accessions from Inner Mongolia were 0.00024 and 0.00045, respectively. There was a high nucleotide diversity. The polymorphism analysis of the *GBSSI* sequence showed (Table 3) that the Hd values of all populations were very high. The population nucleotide diversity of the wild accessions from Shanxi (0.10143) and Heilongjiang (0.12624) in China was significantly higher than that of other wild accessions; Ningxia (0.002200) population had the lowest nucleotide diversity.

**Table 2:** Estimates of genetic diversity between wild accessions and local varieties of broomcorn millet based on *trn*T-*trn*L sequences.

Population	S	Hd	π	θ_w_
**Wild accessions**	1	0.046	0.00006	0.00026
**Local varieties**	10	0.068	0.00007	0.00210

**Table 3:** Estimates of genetic diversity between wild accessions and local varieties of broomcorn millet based on *GBSSI* sequences.

Population	S	Hd	π	θ_w_
**Wild accessions**	302	1.000	0.09145	0.09596
**Local varieties**	399	0.9971	0.11696	0.13476

Overall, the diversity of each population was various in different sequence analysis, indicating that the diversity of sequences varied with different gene types. The Hd values of of *GBSSI* sequences of wild accessions and local varieties were much greater than 0.5, and the π values were much larger than 0.005, indicating that the genetic diversity of the *GBSSI* sequence of broomcorn millet was very high. In comparison, the genetic diversity of *trn*T-*trn*L was lower. In the wild populations, the genetic diversity of resources in Shanxi, Inner Mongolia and Heilongjiang were higher and their genetic resources were abundant.

### 3.3 Neutrality tests

Tajima’s D, Fu and Li’s D*, and Fu and Li’s F* tests can be used to measure the evolution of *trn*T-*trn*L and *GBSSI* in broomcorn millet. At the species level, the Tajima’s D of the *trn*T-*trn*L sequence was significantly negative; Fu and Li’s D* and Fu and Li’s F* values were extremely significantly negative (Table 4), indicating that the diversity of the broomcorn millet *trn*T-*trn*L sequence was a deviation from the neutral evolution pattern. Among them, the Tajima’s D value of the samples from Gansu, China was significant, indicating that negative selection in the long-term evolution of this population. In addition, the population from Gansu was rich in diversity. This is consistent with the results of the sequence polymorphism analysis. Tajima’s D neutrality test of the *GBSSI* sequence at the species level was negative but not significant. At the population level, Tajima’s D of the Shanxi, Inner Mongolia, Liaoning, Heilongjiang, European, and North American populations were all positive, indicating that the broomcorn millet *GBSSI* sequences from these populations underwent positive selection during evolution.

**Table 4:** Neutrality test of 14 broomcorn millet populations.

	*trn*T*-trn*L			*GBSSI*		
Population	Tajima’s D	Fu and Li’s D*	Fu and Li’s F*	Tajima’s D	Fu and Li’s D*	Fu and Li’s F*
**Ningxia, China**	−1.16097	−1.59071	−1.69158	−1.01403	−1.54878	−1.62069
**Shanxi, China**	−1.48074	−1.82689	−1.97405	0.14928	0.34205	0.33216
**Inner Mongolia, China**	−1.48074	−1.82689	−1.97405	0.53000	0.57521	0.65781
**Hebei, China**	−1.13552	−1.73221	−1.80393	−0.24983	0.15759	0.01773
**Liaoning, China**	−1.14915	−0.36547	−1.48111	0.54707	0.85972	0.88825
**Heilongjiang, China**	−1.14244	−1.70335	−1.78196	0.09997	0.40281	0.35480
**Shaanxi, China**				−0.08865	0.26045	0.18870
**Xinjiang, China**	–	–	–	−0.81307	−1.46446	−1.43333
**Gansu, China**	−2.2297^**^	−5.30935^**^	−5.01709^**^	−0.98808	−0.11296	−0.62393
**Asia (excluding China)**	–	–	–	−0.28548	−0.59849	−0.58619
**Europe**	–	–	–	0.24498	0.45867	0.45982
**South America**	–	–	–	–	–	–
**North America**	–	–	–	0.19519	0.26838	0.27613
**Oceania**	–	–	–	−0.02968	−0.01319	−0.01792
**Africa**	–	–	–	–	–	–
**Total**	−2.00208^*^	−5.47430^**^	−5.04885^**^	−0.50680	−0.16788	0.41512

* : Significant, P < 0.05; ** : Extremely significant, P < 0.01

### 3.4 Population genetic similarity and genetic structure analysis

The sequence similarity of *trn*T-*trn*L was high. The intra-species and inter-species genetic distances were 0.000. For the nrDNA *GBSSI* sequences, the genetic distance within the population from Shaanxi, China was the largest (Table 5), indicating Shaanxi population may contain varied originations. This is followed by Heilongjiang population. The genetic distance between wild and local populations ranged from 0.082 to 0.131 (Table 6). The genetic distance between Heilongjiang and the other populations was the largest, all greater than 0.100, indicating that Heilongjiang was genetically distant from the other populations. The genetic distance between local varieties and Hebei wild population was small, indicating that they were genetically closely related.

**Table 5:** Genetic distances within 15 populations based on *GBSSI* sequences.

Population	1	2	3	4	5	6	7	8	9	10	11	12	13	14	15
**Genetic distance**	0.115	0.185	0.185	0.150	0.169	0.191	0.258	0.059	0.094	0.081	0.071	0.063	0.096	0.067	0.084

1: Ningxia, China; 2: Shanxi, China; 3: Inner Mongolia, China; 4: Hebei, China; 5: Liaoning, China; 6: Heilongjiang, China; 7:Shaanxi, China; 8: Xinjiang, China; 9: Gansu, China; 10: Asia (excluding China); 11: Europe; 12: South America; 13: North America; 14: Oceania; 15: Africa

**Table 6:** Genetic distances between wild populations and local varieties based on *GBSSI* sequences.

Populations	NXWP	SXWP	IMWP	HBWP	LNWP	HLJWP	LVP
**NXWP**	–						
**SXWP**	0.089	–					
**IMWP**	0.089	0.093	–				
**HBWP**	0.090	0.088	0.082	–			
**LNWP**	0.092	0.095	0.092	0.085	–		
**HLJWP**	0.131	0.126	0.118	0.119	0.121	–	
**LVP**	0.112	0.114	0.115	0.106	0.112	0.150	–

Note: NXWP is Ningxia wild population, SXWP is Shanxi wild population, IMWP is Inner Mongolia wild population, HBWP is Hebei wild population, LNWP is Liaoning wild population, HLJWP is Heilongjiang wild population, LVP is local varieties population.

The genetic differentiation coefficient (Fst) can be used to assess the distribution of genetic variation within and between populations. Gene flow (Nm) is an important factor affecting genetic variation. The Fst of *trn*T-*trn*L was 0.05041 and the Nm value was 4.71, suggesting that 5.04% of the genetic variation occurred between the populations, and 94.96% of the genetic variation occurred within a population. The genetic differentiation between the populations was weak, indicating intra-population variation is the main source of variation. The Fst value of *GBSSI* was 0.12681, and the Nm value was 1.72, that means, 12.68% of the genetic variation occurred between the populations, and 87.32% of the genetic variation occurred within a population. The intra-population variation is also the main source of variation.

### 3.5 Phylogenetic analysis

**Fig 3:**
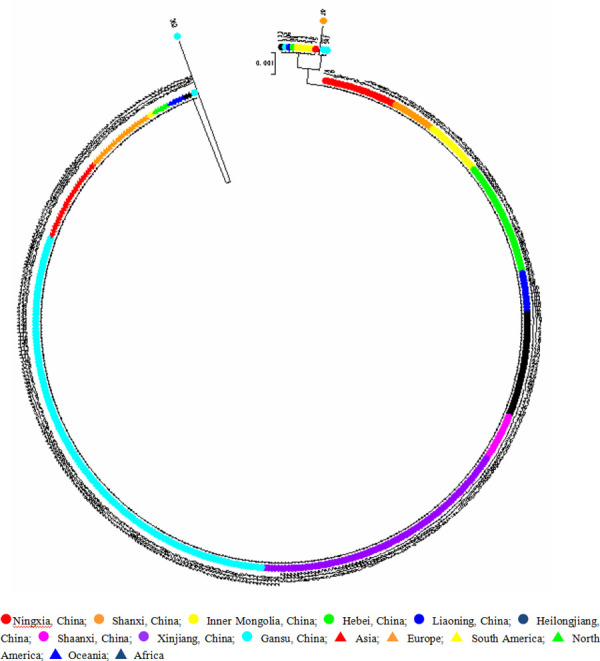
Phylogenetic tree derived from *trn*T*-trn*L using maximum likelihood from broomcorn millet.

Phylogenetic tree was created using the Neighbor-Joining method. Fig 3 shows that the *trn*T-*trn*L sequence of 430 broomcorn millet accessions had little difference, and the region is highly conserved. A majority of accessions were clustered into the same branch, except for individual accessions. The most divergent broomcorn millet accession was Xinghoutoumi from Chongxin, Gansu, indicating that the genetic sequence of this accession is different from other accessions and this assesstion shows high potential as genetic resource as it may contain rare genetic variations. The rest of the resources were grouped into one big branch which could be divided into three groups. The group I consisted of ten resources, including one Ningxia resource, one Hebei resource, one Inner Mongolia resource, five Liaoning resources, one Heilongjiang resource and one Gansu resource. Group II included two resources, both are Gansu resources. Group III consisted of 417 remaining resources. The phylogenetic tree showed that the broomcorn millet has regional characteristics.

**Fig 4:**
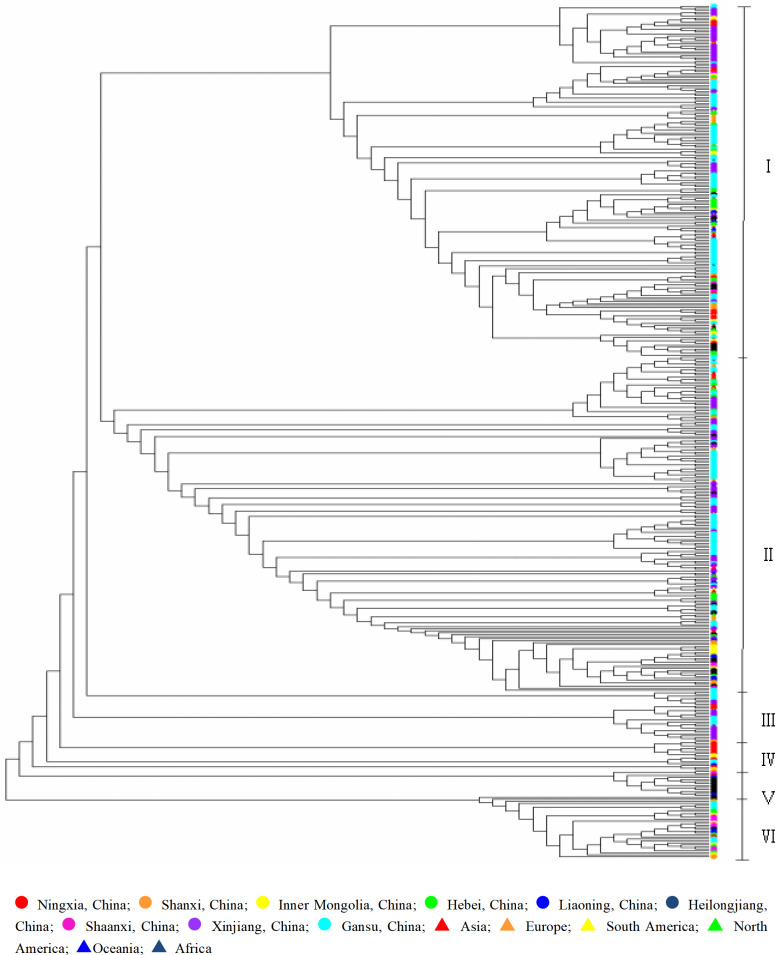
Phylogenetic tree derived from *GBSSI* using maximum likelihood from broomcorn millet.

**Fig 5:**
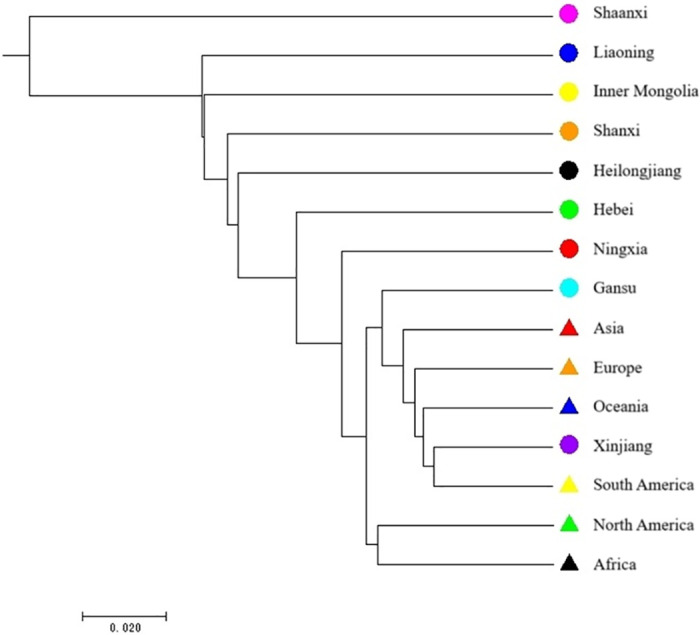
Cluster diagram of broomcorn millet based on *GBSSI* sequence.

Phylogenetic analysis based on the *GBSSI* sequence divided 430 broomcorn millet accessions into six groups (Fig 4), and each group contained accessions from multiple regions. As seen in the dendrogram, most of Ningxia, Shanxi, Inner Mongolia, Hebei, Heilongjiang, Xinjiang, Gansu, Asia (except for China), Europe, North America and Africa accessions were concentrated in group I; half of Shanxi, Hebei, Liaoning, Heilongjiang, Xinjiang, Gansu and Asia, Europe accessions were spread in group II; group III mainly included resources in Ningxia, and a little of Xinjiang and Gansu accessions; group IV mainly included Ningxia and Inner Mongolia accessions; group V contained China’s Heilongjiang accessions and a few Liaoning accessions; some of Hebei, Shaanxi and Gansu accessions were mainly divided into group VI. There was no obvious genetic differentiation between domestic and foreign populations. Foreign and domestic populations may have the same origin. Group IV, group V and group VI were special genotype for domestic resources. Most of the accessions from Ningxia, Shanxi, Inner Mongolia, Hebei, Xinjiang, Gansu, Europe, North America and Africa were distributed into group I. Numerous resources from Liaoning, Heilongjiang, Xinjiang, Gansu, Asia (except for China) and Europe were distributed in group II, indicating that the genetic relationship is close. Ningxia, Shanxi, Inner Mongolia, Liaoning and Gansu accessions were distributed in five groups, indicating that these regions are rich in genetic diversity. As shown in Fig 5, the genetic relationship between the Shaanxi population and the rest of the populations was relatively remote. The genetic relationship of populations in Shanxi, Inner Mongolia, Heilongjiang and Liaoning was close. Hebei, Ningxia, Xinjiang and Gansu had close genetic relationship with foreign populations.

## 4 Discussion

### 4.1 The *trnT-trnL* and *GBSSI* sequence diversity and application

Nucleic acid sequence analysis is beneficial to the study of genetic diversity and the origin of a species. The *trn*T-*trn*L and *GBSSI* sequences are favored by an increasing number of researchers due to their length stability and sequence variability in many fields. In 1991, Taberlet [14] first studied the evolutionary relationship of plants using chloroplast DNA, and introduced the *trn*T-*trn*L sequence into plant phylogeny. Baumel*et al.*[15] used *trn*T-*trn*L, *GBSSI* and *ITS* sequences to analyze the phylogenetics of the *Spartina* Schreb, finding that the degree of variation of nuclear sequences was significantly higher than that of *trn*T-*trn*L, and *GBSSI* had more informative sites and richer genetic diversity. Yang’s research [16] found the genetic diversity of *trn*T-*trn*L was middling, its haplotype diversity index ranged from 0 to 0.900; the nucleotide diversity index ranged from 0 to 0.0544. Hunt *et al.* [17] analyzed phylogeographic structure and the geographic and phylogenetic distribution of the *GBSSI* alleles of 178 broomcorn millet individuals from Eurasia, found that *GBSSI* gene sequence had obvious relationship with spatial distribution. Park *et al*. [18] investigated the genetic diversity of *GBSSI*, 99.7% of the sequences showed very high conservation and low genetic diversity. Based on eight chloroplast non-coding sequence, Meng [13] explored the genetic diversity and phylogenetic relationships of broomcorn millet and its weed forms, he found that the cpDNA non-coding sequences were all relatively conserved. As reported, the genetic diversity of *GBSSI* is a more efficient parameter than that of *trnT-trnL* in exploring evolutionary relationship of plants.

Similarly, in this study, 96.3% sites of *trn*T-*trn*L sequence were conserved. Its genetic diversity was very low. The *GBSSI* sequence diversity of broomcorn millet was significantly more abundant than that of *trn*T-*trn*L, as its diversity parameter was higher than the standard level, which is consistent with the results of Meng and Baumel *et al.*[13,15]. The Fst value showed the main source of variation was within the populations. The nucleotide polymorphisms and haplotype diversity of different populations are also discrepant, indicating sequence diversity varies with species and gene type, it’s coincident with that of Li *et al* [19].

### 4.2 Genetic diversity and relationships of broomcorn millet

There are reports on the genetic diversity and genetic relationship of broomcorn millet at the molecular level. Dong *et al.* [20] used 19 pairs of SSR markers to analyze the genetic diversity of 96 broomcorn millet individuals from Heilongjiang, Inner Mongolia, Ningxia, Shanxi, Shaanxi, and Russia. The results showed that the phylogenetic relationships among the provinces were close and the genetic diversity of accessions from Shanxi was the most abundant. According to the study of Hu et al. [21], Shanxi was also reported as the initial center of origin of broomcorn millet, which then expanded to other regions. In this study, the polymorphism analysis of the two sequences and the Tajima’s D tests all indicated that the genetic diversity of the Gansu population in China was the highest, and the genetic diversity of Shanxi, Inner Mongolia, and Shaanxi populations were also abundant. The core area of broomcorn millet origination may include: Shanxi, Shaanxi, Inner Mongolia, Ningxia and Gansu. After origination and evolution in core area, the broomcorn millet resource may spread westward from Gansu to Xinjiang and enter Eurasia, or eastward from Shanxi to Hebei, Inner Mongolia and northeast China. The phylogenetic analysis based on the GBSSI sequence in this study also supported this potential route, as most of Shanxi, Inner Mongolia, Ningxia, Gansu, Xinjiang, Asia (except for China), Europe, North America and Africa accessions were concentrated in the same cluster group. The Asia (except China), European, Oceania populations showed a close genetic relationship with accessions in northwest China (Ningxia, Xinjiang, Gansu) and north China (Hebei). The results were similar to reports of Wang *et al.* [22] and Xue *et al* [23]*.* Dong *et al.* [24] also reported close phylogenetic relationships among broomcorn millet individuals from Inner Mongolia, Ningxia, Shanxi, Shaanxi, and Russia. Similarly, Zhao [25] and Wang *et al.*[22] evaluated the genetic diversity and population genetic structure of Chinese broomcorn millet accessions, respectively, indicating that the accessions among Shanxi, Ningxia, Gansu, and Inner Mongolia were relatively close, and that of Heilongjiang, Jilin, and Liaoning were related.

### 4.3 Research progress on the origin and spread of broomcorn millet

Broomcorn millet was one of the earliest domesticated crops in the world, and China was widely recognized as the center of origin of the broomcorn millet [26–27]. Previous archaeological data indicated that northwestern China, northern China, and Inner Mongolia were the likely areas of domestication of broomcorn millet [28–30]. In the site of Cishan, the ash layer of broomcorn millet was discovered, which was identified to be about 10,000 years ago, indicating that the middle and lower reaches of the Yellow River might be the origin of broomcorn millet [31]. The Xinglonggou Site in Chifeng, Inner Mongolia, dating from about 8000–7500 years ago, is the earliest found of the broomcorn millet grain remains [21]. Hunt *et al.* [17] supported the argument that northern China was the origin of domestication and early cultivation center by analyzing the geographic origin and phylogenetic of the broomcorn millet *GBSSI* gene in Eurasia. Li *et al.* [10] analyzed the *ITS* and *ETS* sequences of rDNA from the ancient broomcorn millet and the modern local cultivar, he found that the exact sequence matching the broomcorn millet remains dug from Xiaohe cemetery was not the modern local cultivar; the most similar sequence existed in India and Europe. In combination with archaeological records, the authors speculated that the *P. miliaceum* from Xiaohe cemetery began from the Hexi Corridor and passed through northern Xinjiang, Russia, and finally to Eastern Europe and India. Xinjiang is the key corridor for the spread of broomcorn millet. This is similar to the potential route in this study, in which Xinjiang is crucial for broomcorn millet to spread westward. In this study, the genetic diversity within local varieties, northern and northeast China wild populations was close. Northern regions of China may be the origin of broomcorn millet. The genetic relationship among the Eurasian population and the Ningxia, Xinjiang and Gansu populations is close, indicating that the broomcorn millet might have spread westward through the northwestern region of China and entered Eurasia, confirming the research results of Li *et al*. [10]. The available sites for the study of origin evolution are limited, the actual transmission pathway of broomcorn millet needs to be determined by analyses of additional genetic regions.

## 5 Conclusions

In conclusion, our study showed that broomcorn millet may originated from the core area (including Shanxi, Shaanxi, Inner Mongolia, Ningxia and Gansu) and then spread westward to Xinjiang and entered Eurasia, or eastward from Shanxi to Hebei, Inner Mongolia and northeast China. Xinjiang is crucial for broomcorn millet to spread westward. In addition, the *GBSSI* sequence might be an effective molecular marker for the assessment of genetic diversity and exploration of the origin and evolution of broomcorn millet.

## Supporting information

S1 Table The names and sources of broomcorn millet. (ZIP)

## References

[1] Wang RY, Liu XY, Wang HG, Lu P, Liu MX, Chen L. Analysis of genetic diversity of Chinese broomcorn millet by using high elementary microsatellite markers. Scientia Agricultura Sinica. 2017;50(20):3848–59.

[2] Crawford GW. Agricultural origins in North China pushed back to the Pleistocene-Holocene boundary. Proc Natl Acad Sci U S A. 2009;106(18):7271–2. doi: 10.1073/pnas.0903375106 19416918 PMC2678596

[3] Fuller DQ. Agricultural Origins and Frontiers in South Asia: A Working Synthesis. J World Prehist. 2006;20(1):1–86. doi: 10.1007/s10963-006-9006-8

[4] Wang RY. Genetic diversity and evolution advancement in common millet (Panicum miliaceum L.). Beijing: China Agriculture Press; 2017.

[5] Wang XY, Wang L. Proso millet germplasm resources description and panicum standard. Beijing: China Agriculture Press; 2006.

[6] M’ribu HK, Hilu KW. Detection of interspecific and intraspecific variation in Panicum millets through random amplified polymorphic DNA. Theor Appl Genet. 1994;88(3–4):412–6. doi: 10.1007/BF00223653 24186027

[7] Karam D, Westra P, Nissen SJ, Ward SM, Figueiredo JEF. Genetic diversity among proso millet (Panicum miliaceum) biotypes assessed by AFLP technique. Planta daninha. 2004;22(2):167–74. doi: 10.1590/s0100-83582004000200001

[8] Cho Y-I, Chung J-W, Lee G-A, Ma K-H, Dixit A, Gwag J-G, et al. Development and characterization of twenty-five new polymorphic microsatellite markers in proso millet (Panicum miliaceum L.). Genes Genom. 2010;32(3):267–73. doi: 10.1007/s13258-010-0007-8

[9] Sha L-N, Fan X, Li J, Liao J-Q, Zeng J, Wang Y, et al. Contrasting evolutionary patterns of multiple loci uncover new aspects in the genome origin and evolutionary history of Leymus (Triticeae; Poaceae). Mol Phylogenet Evol. 2017;114:175–88. doi: 10.1016/j.ympev.2017.05.015 28533082

[10] Li C, Dong Y, Liu M, Lu P, Li W, Wang Y, et al. Ancient DNA analysis of Panicum miliaceum (broomcorn millet) from a Bronze Age cemetery in Xinjiang, China. Veget Hist Archaeobot. 2016;25(5):469–77. doi: 10.1007/s00334-016-0561-3

[11] Hollingsworth P M, Forrest L L, Spouge J L, Hajibabaei M, Ratnasingham S, Bank M, et al A DNA barcode for land plants. Proc Natl Acad Sci U S A. 2009;106(31):12794–7. doi: 10.1073/pnas.0905845106 19666622 PMC2722355

[12] Aliscioni SS, Giussani LM, Zuloaga FO, Kellogg EA. A molecular phylogeny of Panicum (Poaceae: Paniceae): tests of monophyly and phylogenetic placement within the Panicoideae. Am J Bot. 2003;90(5):796–821. doi: 10.3732/ajb.90.5.796 21659176

[13] Fanshuang M. Studies on population genetics and domestication of *Panicum miliaceum*. Jilin University; 2018.

[14] Taberlet P, Gielly L, Pautou G, Bouvet J. Universal primers for amplification of three non-coding regions of chloroplast DNA. Plant Mol Biol. 1991;17(5):1105–9. doi: 10.1007/BF00037152 1932684

[15] Baumel A, Ainouche ML, Bayer RJ, Ainouche AK, Misset MT. Molecular phylogeny of hybridizing species from the genus Spartina Schreb. (Poaceae). Mol Phylogenet Evol. 2002;22(2):303–14. doi: 10.1006/mpev.2001.1064 11820850

[16] Yang LC, Liu CH, Zhou XL, Xu WH, Zhou GY. Polymorphism analysis of endangered Notopterygium incisum and endemic species based on cpDNA trnT-trnL sequences. Chinese Traditional and Herbal Drugs. 2015;46(22):3390–5.

[17] Hunt HV, Moots HM, Graybosch RA, Jones H, Parker M, Romanova O, et al. Waxy phenotype evolution in the allotetraploid cereal broomcorn millet: mutations at the GBSSI locus in their functional and phylogenetic context. Mol Biol Evol. 2012;30(1):109–22. doi: 10.1093/molbev/mss209 22936718 PMC3533377

[18] Park Y-J, Nemoto K, Nishikawa T, Matsushima K, Minami M, Kawase M. Genetic diversity and expression analysis of granule bound starch synthase I gene in the new world grain amaranth (Amaranthus cruentus L.). Journal of Cereal Science. 2011;53(3):298–305. doi: 10.1016/j.jcs.2011.01.011

[19] Li M, Zhang ZW, Li YQ, Wu B, Gao J. Genetic diversity of Tartary Buckwheat based on the sequence analysis of PAL. Journal of Plant Genetic Resources. 2017;18(3):530–7.

[20] Dong JL, Wang HG, Chen L, Wang JJ, Cao XN, Wang L. Analysis of genetic diversity and genetic structure of broomcorn millet of the Chinese. Scientia Agricultura Sinica. 2015;48(16):3121–31.

[21] Hu X, Wang J, Lu P, Zhang H. Assessment of genetic diversity in broomcorn millet (Panicum miliaceum L.) using SSR markers. J Genet Genomics. 2009;36(8):491–500. doi: 10.1016/S1673-8527(08)60139-3 19683672

[22] Wang RY, Ji X, Lu P, Liu MX, Xu Y, Wang L, et al. Analysis of genetic diversity of Chinese broomcorn millet by fluorescence SSR. Acta Agron Sin. 2017;43(4):530–48.

[23] Xue YT, Lu P, Qiao ZJ, Liu MX, Wang RY. Genetic diversity and genetic telationship of broomcorn millet (Panicum miliaceum L.) germplasm based on SSR markers. Scientia Agricultura Sinica. 2018;51(15):19–39.

[24] Dong YC, Zheng DS. Crops and Their Wild Relatives in China: Food Crops Rolls. Beijing: China Agriculture Press; 2006.

[25] Zhao FL. Genetic diversity of broomcorn millet from different ecotype zone in China based on SSR. Shanxi Agricultural University; 2014.

[26] Rajput SG, Santra DK. Evaluation of Genetic Diversity of Proso Millet Germplasm Available in the United States using Simple‐Sequence Repeat Markers. Crop Science. 2016;56(5):2401–9. doi: 10.2135/cropsci2015.10.0644

[27] Wei YH. On the origin of broomcorn millet. Agricultural Archeology. 1986;2:248–51.

[28] Bettinger RL, Barton L, Morgan C. The origins of food production in north China: a different kind of agricultural revolution. EvolAnthropol. 2010;19:9–21.

[29] Lu H, Zhang J, Liu K, Wu N, Li Y, Zhou K, et al. Earliest domestication of common millet (Panicum miliaceum) in East Asia extended to 10,000 years ago. Proc Natl Acad Sci U S A. 2009;106(18):7367–72. doi: 10.1073/pnas.0900158106 19383791 PMC2678631

[30] Miller NF, Spengler RN, Frachetti M. Millet cultivation across Eurasia: Origins, spread, and the influence of seasonal climate. Holocene. 2016;26(10):1566–75. doi: 10.1177/0959683616641742

[31] Lv H Y, Zhang J P, Liao G B, Wu N Q, Li Y M, Zhou K S, et al. Analysis of siliceous body of millet and the origin of East Asian dry farming. The 8th Annual Academic Meeting of the Chinese Paleontological Palynology Branch. 2009, 1 p.

