# Supplementary material for: Genetic diversity and relationships of broomcorn millet based on trnT-trnL and GBSSI sequences
Source: PLoS One. 2025 Jul 23;20(7):e0325433. doi: 10.1371/journal.pone.0325433 (PMC12286405; doi:10.1371/journal.pone.0325433)
Supplement: S1 Table — (ZIP) [file pone.0325433.s001.zip › Supporting information/Table S1 The names and sources of broomcorn millet.docx]

Table S1 The names and sources of broomcorn millet

| Number | Group number and name | Material name | Material source |
| --- | --- | --- | --- |
| 1 | 1 Ningxia | Ningxia-1 | Ningxia |
| 2 | Ningxia | Ningxia-2 | Ningxia |
| 3 | Ningxia | Ningxia-3 | Ningxia |
| 4 | Ningxia | Ningxia-4 | Ningxia |
| 5 | Ningxia | Ningxia-5 | Ningxia |
| 6 | Ningxia | Ningxia-6 | Ningxia |
| 7 | Ningxia | Ningxia-7 | Ningxia |
| 8 | Ningxia | Ningxia-8 | Ningxia |
| 9 | Ningxia | Ningxia-9 | Ningxia |
| 10 | Ningxia | Ningxia-10 | Ningxia |
| 11 | Ningxia | Ningxia-11 | Ningxia |
| 12 | Ningxia | Ningxia-12 | Ningxia |
| 13 | Ningxia | Ningxia-13 | Ningxia |
| 14 | Ningxia | Ningxia-14 | Ningxia |
| 15 | Ningxia | Ningxia-15 | Ningxia |
| 16 | Ningxia | Huinonghuangnianshu | Yingluo, Ningxia |
| 17 | Ningxia | Huaruanmizi | Yanchi, Nimgxia |
| 18 | Ningxia | Helanerhuang | Helan, Nimgxia |
| 19 | Ningxia | Baimizi | Yongning, Ningxia |
| 20 | Ningxia | 66-3-98 | Yongning, Ningxia |
| 21 | Ningxia | Jinjidahuangmi | Wuzhong, Ningxia |
| 22 | Ningxia | Yandongshanzigan | Yanchi, Nimgxia |
| 23 | Ningxia | Misuihong | Zhongwei, Ningxia |
| 24 | 2Shanxi | Datong-1 | Datong |
| 25 | Shanxi | Datong-2 | Datong |
| 26 | Shanxi | Shanxigangxianyemizi | Gangxian, Shanxi |
| 27 | Shanxi | Yuza1 | Baode, Shanxi |
| 28 | Shanxi | Huangmizi | Yuanping, Shanxi |
| 29 | Shanxi | Xiaoqingmizi | Wuzhai, Shanxi |
| 30 | Shanxi | Huangluosan | Ningwu, Shanxi |
| 31 | Shanxi | Huimizi | Loufan, Shanxi |
| 32 | Shanxi | Shuzi | Shouyang, Shanxi |
| 33 | Shanxi | Dahuangshu | Heshun, Shanxi |
| 34 | Shanxi | Huami | Yanggao, Shanxi |
| 35 | Shanxi | Yemi | Yanggao, Shanxi |
| 36 | Shanxi | Baimizi | Shuo county, Shanxi |
| 37 | Shanxi | Xiaohongmi | Datong, Shanxi |
| 38 | 3 Inner Mongolia | Chifeng-2 | Chifeng, Inner Mongolia |
| 39 | Inner Mongolia | Chifeng-4 | Chifeng, Inner Mongolia |
| 40 | Inner Mongolia | Chifeng-5 | Chifeng, Inner Mongolia |
| 41 | Inner Mongolia | Chifeng-6 | Chifeng, Inner Mongolia |
| 42 | Inner Mongolia | Chifeng-7 | Chifeng, Inner Mongolia |
| 43 | Inner Mongolia | Chifeng-8 | Chifeng, Inner Mongolia |
| 44 | Inner Mongolia | Neimeng-2 | Inner Mongolia |
| 45 | Inner Mongolia | Neimeng-3 | Inner Mongolia |
| 46 | Inner Mongolia | Neimeng-4 | Inner Mongolia |
| 47 | Inner Mongolia | Neimeng-5 | Inner Mongolia |
| 48 | Inner Mongolia | Neimeng-6 | Inner Mongolia |
| 49 | Inner Mongolia | Humenglishuzi | Hu League, Inner Mongolia |
| 50 | Inner Mongolia | Zuozhongqigaoliangmi | Zhe League, Inner Mongolia |
| 51 | Inner Mongolia | Aqigaoliangmi | Zhao League, Inner Mongolia |
| 52 | Inner Mongolia | Qingshuihedabaishu | Wu League, Inner Mongolia |
| 53 | Inner Mongolia | Wuyuanheishuzi | Ba League, Inner Mongolia |
| 54 | Inner Mongolia | Hanghouxiaoqingmi | Ba League, Inner Mongolia |
| 55 | Inner Mongolia | Ba826huangshuzi | Ba League, Inner Mongolia |
| 56 | Inner Mongolia | Bamenghuangshuzi | Ba League, Inner Mongolia |
| 57 | Inner Mongolia | Daqidabaishuzi | Yi League, Inner Mongolia |
| 58 | Inner Mongolia | Wuyuanyidianqing | Ba League, Inner Mongolia |
| 59 | Inner Mongolia | Linheshuanglishu | Ba League, Inner Mongolia |
| 60 | 4 Hebei | Chengde-2 | Chengde,Hebei |
| 61 | Hebei | Chengde-3 | Chengde,Hebei |
| 62 | Hebei | Chengde-5 | Chengde,Hebei |
| 63 | Hebei | Chengde-A | Chengde,Hebei |
| 64 | Hebei | Chengde-6 | Chengde,Hebei |
| 65 | Hebei | Chengde-7 | Chengde,Hebei |
| 66 | Hebei | Chengde-8 | Chengde,Hebei |
| 67 | Hebei | Chengde-9 | Chengde,Hebei |
| 68 | Hebei | Chengde B | Chengde,Hebei |
| 69 | Hebei | Chengde-10 | Chengde,Hebei |
| 70 | Hebei | Chengde-11 | Chengde,Hebei |
| 71 | Hebei | Chengde C | Chengde,Hebei |
| 72 | Hebei | Chengde-12 | Chengde,Hebei |
| 73 | Hebei | Chengde D | Chengde,Hebei |
| 74 | Hebei | Hebei-4 | Hebei |
| 75 | Hebei | Hebei-5 | Hebei |
| 76 | Hebei | Hebei-6 | Hebei |
| 77 | Hebei | Hebei-9 | Hebei |
| 78 | Hebei | Hebei-10 | Hebei |
| 79 | Hebei | Hebei-14 | Hebei |
| 80 | Hebei | Hebei-15 | Hebei |
| 81 | Hebei | Hebei-16 | Hebei |
| 82 | Hebei | Hebei-17 | Hebei |
| 83 | Hebei | Hebei-19 | Hebei |
| 84 | Hebei | Hebei-20 | Hebei |
| 85 | Hebei | Hebei-7 | Hebei |
| 86 | Hebei | Baizilaidai | Kangbao, Hebei |
| 87 | Hebei | Xiaoshuzi | Chongli, Hebei |
| 88 | Hebei | Zhaolitou | Chengde, Hebei |
| 89 | Hebei | Dazigan | Yu county, Hebei |
| 90 | Hebei | Gudubai | Yu county, Hebei |
| 91 | Hebei | Heimizi | Pingshan, Hebei |
| 92 | Hebei | Heikeshu | Shen county, Hebei |
| 93 | Hebei | Nianmizi | Xiong county, Hebei |
| 94 | Hebei | Gaoliangshu | Shulu, Hebei |
| 95 | 5 Liaoning | Liaoning-1 | Liaoning |
| 96 | Liaoning | Liaoning-2 | Liaoning |
| 97 | Liaoning | Liaoning-3 | Liaoning |
| 98 | Liaoning | Liaoning-6 | Liaoning |
| 99 | Liaoning | Liaoning-7 | Liaoning |
| 100 | Liaoning | Liaoning-8 | Liaoning |
| 101 | Liaoning | Liaoning-9 | Liaoning |
| 102 | Liaoning | Mizi | Montenegro, Liaoning |
| 103 | Liaoning | Huangmizi | Liaozhong, Liaoning |
| 104 | Liaoning | Dahongshu | Chaoyang, Liaoning |
| 105 | Liaoning | Dabaishu | Chaoyang, Liaoning |
| 106 | Liaoning | Mazhayan | Sea area, Liaoning |
| 107 | Liaoning | Jinxianhuangmizi | Jin county, Liaoning |
| 108 | 6 Heilongjiang | Qiqihaerye-1 | Qiqihar,Heilongjiang |
| 109 | Heilongjiang | Qiqihaerye-2 | Qiqihar,Heilongjiang |
| 110 | Heilongjiang | Qiqihaerye-3 | Qiqihar,Heilongjiang |
| 111 | Heilongjiang | Qiqihaerye-4 | Qiqihar,Heilongjiang |
| 112 | Heilongjiang | Qiqihaerye-5 | Qiqihar,Heilongjiang |
| 113 | Heilongjiang | Qiqihaerye-6 | Qiqihar,Heilongjiang |
| 114 | Heilongjiang | Qiqihaerye-7 | Qiqihar,Heilongjiang |
| 115 | Heilongjiang | Heilongjiang245-1 | Heilongjiang |
| 116 | Heilongjiang | Heilongjiang245-2 | Heilongjiang |
| 117 | Heilongjiang | Heilongjiang245-3 | Heilongjiang |
| 118 | Heilongjiang | Heilongjiang245-4 | Heilongjiang |
| 119 | Heilongjiang | Heilongjiang245-5 | Heilongjiang |
| 120 | Heilongjiang | Heilongjiang245-6 | Heilongjiang |
| 121 | Heilongjiang | Heilongjiang245-7 | Heilongjiang |
| 122 | Heilongjiang | Heilongjiang246-2 | Heilongjiang |
| 123 | Heilongjiang | Heilongjiang246-3 | Heilongjiang |
| 124 | Heilongjiang | Heilongjiang246-4 | Heilongjiang |
| 125 | Heilongjiang | Heilongjiang246-5 | Heilongjiang |
| 126 | Heilongjiang | Heilongjiang246-6 | Heilongjiang |
| 127 | Heilongjiang | Heilongjiang246-8 | Heilongjiang |
| 128 | Heilongjiang | Heilongjiang247-1 | Heilongjiang |
| 129 | Heilongjiang | Heilongjiang247-2 | Heilongjiang |
| 130 | Heilongjiang | Heilongjiang248-1 | Heilongjiang |
| 131 | Heilongjiang | Heimizi | Na r, Heilongjiang |
| 132 | Heilongjiang | Shuanglimizi | Kedong, Heilongjiang |
| 133 | Heilongjiang | Dahongmizi | Heilongjiang |
| 134 | Heilongjiang | Huangmizi | Rich brocade, Heilongjiang |
| 135 | Heilongjiang | Baimizi | Huachuan, Heilongjiang |
| 136 | Heilongjiang | Maimizi | Mishan, Heilongjiang |
| 137 | Heilongjiang | Xiaomaimizi | Linkou, Heilongjiang |
| 138 | Heilongjiang | Heimizi | Dongning, Heilongjiang |
| 139 | Heilongjiang | Mizi | Shuangcheng, Heilongjiang |
| 140 | 7 Shaanxi | Huimizi | Fugu, Shaanxi |
| 141 | Shaanxi | Ziganhongshu | Shenmu, Shaanxi |
| 142 | Shaanxi | Dawahui | Yulin, Shaanxi |
| 143 | Shaanxi | Niuluandanmi | Jingbian, Shaanxi |
| 144 | Shaanxi | Bairuanmi | Dingbian, Shaanxi |
| 145 | Shaanxi | Baisansanmi | Zizhou, Shaanxi |
| 146 | Shaanxi | Gedaruanmi | Qingjian, Shaanxi |
| 147 | Shaanxi | Dawahui | Zichang, Shaanxi |
| 148 | Shaanxi | Maruanmi | Zhidan, Shaanxi |
| 149 | Shaanxi | Hongruanmi | Wuqi, Shaanxi |
| 150 | Shaanxi | Xiaohongruanmi | Yanan, Shaanxi |
| 151 | Shaanxi | Saozhouruanmi | Yichuan, Shaanxi |
| 152 | Shaanxi | Hongruanmi | Huanglong, Shaanxi |
| 153 | Shaanxi | Hongmi | Dali, Shaanxi |
| 154 | 8 Xinjiang | Hongmi | Qinghe, Xinjiang |
| 155 | Xinjiang | Huangmi | Qinghe, Xinjiang |
| 156 | Xinjiang | Huangmi | Qinghe, Xinjiang |
| 157 | Xinjiang | Mizi | Qinghe, Xinjiang |
| 158 | Xinjiang | Zamizi | Qinghe, Xinjiang |
| 159 | Xinjiang | Mizi | Aletai, Xinjiang |
| 160 | Xinjiang | Hongmizi | Habahe, Xinjiang |
| 161 | Xinjiang | Huangmizi | Habahe, Xinjiang |
| 162 | Xinjiang | Mizi | Habahe, Xinjiang |
| 163 | Xinjiang | Mizi | Habahe, Xinjiang |
| 164 | Xinjiang | Huangmizi | Jeminay, Xinjiang |
| 165 | Xinjiang | Hongmi | Tarbagatay, Xinjiang |
| 166 | Xinjiang | Hongmi | Tarbagatay, Xinjiang |
| 167 | Xinjiang | Hongmi | Tarbagatay, Xinjiang |
| 168 | Xinjiang | Huangmi | Tarbagatay, Xinjiang |
| 169 | Xinjiang | Huangmi | Tarbagatay, Xinjiang |
| 170 | Xinjiang | Huangmi | Tarbagatay, Xinjiang |
| 171 | Xinjiang | Huangzi | Tarbagatay, Xinjiang |
| 172 | Xinjiang | Huangmi | Tarbagatay, Xinjiang |
| 173 | Xinjiang | Mizi | Tarbagatay, Xinjiang |
| 174 | Xinjiang | Mizi | Tarbagatay, Xinjiang |
| 175 | Xinjiang | Mizi | Tarbagatay, Xinjiang |
| 176 | Xinjiang | Zamizi | Tarbagatay, Xinjiang |
| 177 | Xinjiang | Eminhongmi | Emin, Xinjiang |
| 178 | Xinjiang | Eminhuangmi | Emin, Xinjiang |
| 179 | Xinjiang | Mizi | Yiwu, Xinjiang |
| 180 | Xinjiang | Mizi | Balikun, Xinjiang |
| 181 | Xinjiang | Mizi | Balikun, Xinjiang |
| 182 | Xinjiang | Mizi | Hami, Xinjiang |
| 183 | Xinjiang | Huangmizi | Urumqi, Xinjiang |
| 184 | Xinjiang | Mizi | Urumqi, Xinjiang |
| 185 | Xinjiang | Huangmizi | Fukang, Xinjiang |
| 186 | Xinjiang | Huamizi | Fukang, Xinjiang |
| 187 | Xinjiang | Baimizi | Miquan, Xinjiang |
| 188 | Xinjiang | Mizi | Miquan, Xinjiang |
| 189 | Xinjiang | Huangmi | Changji, Xinjiang |
| 190 | Xinjiang | Huangmi | Changji, Xinjiang |
| 191 | Xinjiang | Huangmi | Changji, Xinjiang |
| 192 | Xinjiang | Mizi | Changji, Xinjiang |
| 193 | Xinjiang | Mizi | Changji, Xinjiang |
| 194 | Xinjiang | Huangmizi | Manas, Xinjiang |
| 195 | Xinjiang | Hongmi | Shawan, Xinjiang |
| 196 | Xinjiang | Hongmi | Shawan, Xinjiang |
| 197 | Xinjiang | Hongmi | Shawan, Xinjiang |
| 198 | Xinjiang | Hongmi | Shawan, Xinjiang |
| 199 | Xinjiang | Hongmizi | Shawan, Xinjiang |
| 200 | Xinjiang | Hongmi | Shawan, Xinjiang |
| 201 | Xinjiang | Huangmi | Shawan, Xinjiang |
| 202 | Xinjiang | Mi | Shawan, Xinjiang |
| 203 | Xinjiang | Huangmi | Shawan, Xinjiang |
| 204 | Xinjiang | Huangmi | Shawan, Xinjiang |
| 205 | Xinjiang | Huangmi | Shawan, Xinjiang |
| 206 | Xinjiang | Mizi | Shawan, Xinjiang |
| 207 | Xinjiang | Mizi | Shawan, Xinjiang |
| 208 | Xinjiang | Mizi | Shawan, Xinjiang |
| 209 | Xinjiang | Hongmizi | Xinyuan, Xinjiang |
| 210 | Xinjiang | Hongmizi | Xinyuan, Xinjiang |
| 211 | Xinjiang | Hongmi | Tekes, Xinjiang |
| 212 | Xinjiang | Heimizi | Tekes, Xinjiang |
| 213 | Xinjiang | Heimizi | Tekes, Xinjiang |
| 214 | Xinjiang | Hongmi | Yining, Xinjiang |
| 215 | Xinjiang | Hongmi | Yining, Xinjiang |
| 216 | Xinjiang | Huangmi | Yining, Xinjiang |
| 217 | Xinjiang | Huangmi | Yining, Xinjiang |
| 218 | Xinjiang | Baimi | Yining, Xinjiang |
| 219 | Xinjiang | Mizi | Yining, Xinjiang |
| 220 | Xinjiang | Mizi | Xinjiang |
| 221 | Xinjiang | Huangmizi | Huocheng, Xinjiang |
| 222 | Xinjiang | Huangmizi | Yanqi, Xinjiang |
| 223 | Xinjiang | Mizi | Yanqi, Xinjiang |
| 224 | Xinjiang | Huangmi | Korla, Xinjiang |
| 225 | Xinjiang | Mizi | Korla, Xinjiang |
| 226 | Xinjiang | Mi | Baicheng, Xinjiang |
| 227 | Xinjiang | Yemizi | Xinjiang |
| 228 | Xinjiang | Tulufanmi | Xinjiang |
| 229 | Xinjiang | Shulefumi | Xinjiang |
| 230 | Xinjiang | A85-60 | Xinjiang |
| 231 | 9 Gansu | Gaolanyadanqing | Gaolan, Gansu |
| 232 | Gansu | Jingchuanxiaobaimi | Jingchuan, Gansu |
| 233 | Gansu | Lingtaihongnianmi | Lingtai, Gansu |
| 234 | Gansu | Lingtaijidanpi | Lingtai, Gansu |
| 235 | Gansu | Pingliangdahongmi | Pingliang, Gansu |
| 236 | Gansu | Huatingnuomizi | Huating, Gansu |
| 237 | Gansu | Jingninghongranmi | Jingning, Gansu |
| 238 | Gansu | Dunhuangheimizi | Dunhuang, Xinjiang |
| 239 | Gansu | Dunhuanghuimizi | Dunhuang, Xinjiang |
| 240 | Gansu | Jintaliuhuangdamizi | Jinta, Gansu |
| 241 | Gansu | Jintajijinpibaizi | Jinta, Gansu |
| 242 | Gansu | Jintahuangzi | Jinta, Gansu |
| 243 | Gansu | Jintadahuangmizi | Jinta, Gansu |
| 244 | Gansu | Jintadaheimi | Jinta, Gansu |
| 245 | Gansu | Jiuquanxiaohongmizi | Jiuquan, Gansu |
| 246 | Gansu | Jiuquanxiaohuangmizi | Jiuquan, Gansu |
| 247 | Gansu | Jinquandahuangmizi | Jiuquan, Gansu |
| 248 | Gansu | Jiuquanheimizi | Jiuquan, Gansu |
| 249 | Gansu | Gaotaiwumizi | Gaotai, Gansu |
| 250 | Gansu | Linzeheidamizi | Linze, Gansu |
| 251 | Gansu | Linzemizi | Linze, Gansu |
| 252 | Gansu | Zhangyelaohuangmizi | Zhangye, Gansu |
| 253 | Gansu | Zhangyedahuangmizi | Zhangye, Gansu |
| 254 | Gansu | Minlehongmizi | Minle, Gansu |
| 255 | Gansu | Minqindahongmizi | Minqin, Gansu |
| 256 | Gansu | Minqinxiaohuangmizi | Minqin, Gansu |
| 257 | Gansu | Minqindahuangmizi | Minqin, Gansu |
| 258 | Gansu | Minqindabaimizi | Minqin, Gansu |
| 259 | Gansu | Minqingedatoubaimizi | Minqin, Gansu |
| 260 | Gansu | Minqinxiaoheimizi | Minqin, Gansu |
| 261 | Gansu | Wuweidahongmizi | Wuwei, Gansu |
| 262 | Gansu | Wuweidahuangmizi | Wuwei, Gansu |
| 263 | Gansu | Wuwei60huangheimizi | Wuwei, Gansu |
| 264 | Gansu | Gulangdahongmizi | Gulang, Gansu |
| 265 | Gansu | Gulanghonggeda | Gulang, Gansu |
| 266 | Gansu | Gulangdahongmizi | Gulang, Gansu |
| 267 | Gansu | Gulang60huangmizi | Gulang, Gansu |
| 268 | Gansu | Gulangheimizi | Gulang, Gansu |
| 269 | Gansu | Gulangbangehong | Gulang, Gansu |
| 270 | Gansu | Gulangbangehong | Gulang, Gansu |
| 271 | Gansu | Yongdenghongmi | Yongdeng, Gansu |
| 272 | Gansu | Yongdengxiaohuangmi | Yongdeng, Gansu |
| 273 | Gansu | Yongdengziganhuangmi | Yongdeng, Gansu |
| 274 | Gansu | Yongdengxiaohuangmi | Yongdeng, Gansu |
| 275 | Gansu | Yongdengxiaoheimi | Yongdeng, Gansu |
| 276 | Gansu | Gaolanhongmi | Gaolan, Gansu |
| 277 | Gansu | Gaolandahongmi | Gaolan, Gansu |
| 278 | Gansu | Gaolandahongmi | Gaolan, Gansu |
| 279 | Gansu | Gaolanxiaohongmi | Gaolan, Gansu |
| 280 | Gansu | Gaolanhuangmizi | Gaolan, Gansu |
| 281 | Gansu | Gaolanxiaohuangmi | Gaolan, Gansu |
| 282 | Gansu | Gaolandahuangmi | Gaolan, Gansu |
| 283 | Gansu | Gaolandahuangmi | Gaolan, Gansu |
| 284 | Gansu | Gaolanzicaohuangmizi | Gaolan, Gansu |
| 285 | Gansu | Gaolanxiaohuangmizi | Gaolan, Gansu |
| 286 | Gansu | Gaolanzigaitou | Gaolan, Gansu |
| 287 | Gansu | Gaolandabaimi | Gaolan, Gansu |
| 288 | Gansu | Gaolanjidanpi | Gaolan, Gansu |
| 289 | Gansu | Gaolanbaimi | Gaolan, Gansu |
| 290 | Gansu | Gaolanbaimi | Gaolan, Gansu |
| 291 | Gansu | Gaolanbaimi | Gaolan, Gansu |
| 292 | Gansu | Gaolanbaimi | Gaolan, Gansu |
| 293 | Gansu | Gaolanbaimi | Gaolan, Gansu |
| 294 | Gansu | Gaolanxiaobaimizi | Gaolan, Gansu |
| 295 | Gansu | Gaolandabaimizi | Gaolan, Gansu |
| 296 | Gansu | Gaolanjidanpi | Gaolan, Gansu |
| 297 | Gansu | Gaolanjidanpi | Gaolan, Gansu |
| 298 | Gansu | Gaolanyadanpi | Gaolan, Gansu |
| 299 | Gansu | Gaolanyadanqing | Gaolan, Gansu |
| 300 | Gansu | Qinggaolanxiaoheimi | Gaolan, Gansu |
| 301 | Gansu | Gaolanjidanqing | Gaolan, Gansu |
| 302 | Gansu | Gaolandaqingmizi | Gaolan, Gansu |
| 303 | Gansu | Gaolanbanlianhong | Gaolan, Gansu |
| 304 | Gansu | Lanzhouxiaohuangmizi | Lanzhou, Gansu |
| 305 | Gansu | Baiyinwuyanhuangmi | Lanzhou, Gansu |
| 306 | Gansu | Yuzhongxiaohongmi | Yuzhong, Gansu |
| 307 | Gansu | Yuzhongxiaojinhuang | Yuzhong, Gansu |
| 308 | Gansu | Yuzhongxiaohuangmi | Yuzhong, Gansu |
| 309 | Gansu | Yuzhongdajinhuang | Yuzhong, Gansu |
| 310 | Gansu | Yuzhonghuangmi | Yuzhong, Gansu |
| 311 | Gansu | Yuzhonghongmizi | Yuzhong, Gansu |
| 312 | Gansu | Yuzhongdabaimi | Yuzhong, Gansu |
| 313 | Gansu | Yuzhongheimizi | Yuzhong, Gansu |
| 314 | Gansu | Yuzhongxiaoheimizi | Yuzhong, Gansu |
| 315 | Gansu | Jingchuanxiaohongmi | Jingchuan, Gansu |
| 316 | Gansu | Jingchuanhongnianmi | Jingchuan, Gansu |
| 317 | Gansu | Jingchuanerhanmi | Jingchuan, Gansu |
| 318 | Gansu | Jingchuanhuangxinghoutou | Jingchuan, Gansu |
| 319 | Gansu | Jingchuanqieganmi | Jingchuan, Gansu |
| 320 | Gansu | Jingchuanhuangnuoxiaomi | Jingchuan, Gansu |
| 321 | Gansu | Jingchuanheinianmi | Jingchuan, Gansu |
| 322 | Gansu | Jingchuanheinuoxiaomi | Jingchuan, Gansu |
| 323 | Gansu | Lingtaihongmizi | Lingtai, Gansu |
| 324 | Gansu | Lingtai60huangmi | Lingtai, Gansu |
| 325 | Gansu | Lingtaihuangyingmi | Lingtai, Gansu |
| 326 | Gansu | Lingtainiuweichuan | Lingtai, Gansu |
| 327 | Gansu | Lingtaiqimi | Lingtai, Gansu |
| 328 | Gansu | Chongxindahongmi | Chongxin, Gansu |
| 329 | Gansu | Chongxinxinghoutou | Chongxin, Gansu |
| 330 | Gansu | Chongxinbaidami | Chongxin, Gansu |
| 331 | Gansu | Chongxinjidanpi | Chongxin, Gansu |
| 332 | Gansu | Pingliangxinghoutou | Pingliang, Gansu |
| 333 | Gansu | Pinglianghongmi | Pingliang, Gansu |
| 334 | Gansu | Pingliangzhaolitou | Pingliang, Gansu |
| 335 | Gansu | Pinglianghongdami | Pingliang, Gansu |
| 336 | Gansu | Pingliang80tianhongmizi | Pingliang, Gansu |
| 337 | Gansu | Pinglianghongnianmi | Pingliang, Gansu |
| 338 | Gansu | Pingliang60tianzhaolitou | Pingliang, Gansu |
| 339 | Gansu | Pingliang60tianheixiaomi | Pingliang, Gansu |
| 340 | Gansu | Huatinghongmi | Huating, Gansu |
| 341 | Gansu | Huatinghuangmi | Huating, Gansu |
| 342 | Gansu | Huatingzimi | Huating, Gansu |
| 343 | Gansu | Huatingheimizi | Huating, Gansu |
| 344 | Gansu | Huatingmamizi | Huating, Gansu |
| 345 | Gansu | Zhuanglanglaohuangmi | Zhuanglang, Gansu |
| 346 | Gansu | Zhuanglangxiaoqingmi | Zhuanglang, Gansu |
| 347 | Gansu | Jingningxiaozimi | Jingning, Gansu |
| 348 | Gansu | Jingningxiaohuangmi | Jingning, Gansu |
| 349 | Gansu | Jingningguduimi | Jingning, Gansu |
| 350 | Gansu | Jingningdahuangmi | Jingning, Gansu |
| 351 | Gansu | Jingningdongbeimi | Jingning, Gansu |
| 352 | Gansu | Jingningxiaohuangmi | Jingning, Gansu |
| 353 | Gansu | Jingningsalihuang | Jingning, Gansu |
| 354 | Gansu | Jingningheimizi | Jingning, Gansu |
| 355 | Gansu | Jingningdamami | Jingning, Gansu |
| 356 | Gansu | Jingningxiaoheimi | Jingning, Gansu |
| 357 | Gansu | Minqingedatoumi | Minqin, Gansu |
| 358 | Gansu | Minqinhongmi | Gansu |
| 359 | Gansu | Anximi | Gansu |
| 360 | Gansu | Gaotaimi | Gansu |
| 361 | Gansu | Linzeheidamizi | Gansu |
| 362 | Gansu | Chongxinxinghoutoumi | Chongxin, Gansu |
| 363 | Gansu | Chongxin60tianmi | Chongxin, Gansu |
| 364 | Gansu | Chongxinbaidami | Chongxin, Gansu |
| 365 | Gansu | Jingchuanerhanmi | Jingchuan, Gansu |
| 366 | Gansu | Gulangbangehong | Gulang, Gansu |
| 367 | Gansu | Dunhuangdamizi | Dunhuang, Xinjiang |
| 368 | Gansu | Minqindahuangmizi | Minqin, Gansu |
| 369 | Gansu | Jingchuanxiaohongmi | Jingchuan, Gansu |
| 370 | 10 Asia | AMES32315 | Georgia |
| 371 | Asia | AMES32316 | Georgia |
| 372 | Asia | PI163298 | India |
| 373 | Asia | PI163300 | India |
| 374 | Asia | PI170592 | Turkey |
| 375 | Asia | PI207501 | Afghanistan |
| 376 | Asia | PI211058 | Afghanistan |
| 377 | Asia | PI211059 | Afghanistan |
| 378 | Asia | PI211060 | Afghanistan |
| 379 | Asia | PI212108 | Afghanistan |
| 380 | Asia | PI202317 | Afghanistan |
| 381 | Asia | PI253953 | Afghanistan |
| 382 | Asia | PI253955 | Afghanistan |
| 383 | Asia | PI649381 | Afghanistan |
| 384 | Asia | PI269955 | Pakistan |
| 385 | Asia | PI269957 | Pakistan |
| 386 | Asia | PI269958 | Pakistan |
| 387 | Asia | PI269959 | Pakistan |
| 388 | Asia | PI346939 | Kazakhstan |
| 389 | Asia | PI427247 | Nepal |
| 390 | Asia | PI427247 | Nepal |
| 391 | Asia | PI427248 | Nepal |
| 392 | Asia | PI427249 | Nepal |
| 393 | Asia | PI427250 | Nepal |
| 394 | Asia | PI433381 | Taiwan |
| 395 | 11 Europe | PI649372 | France |
| 396 | Europe | PI477123 | Germany |
| 397 | Europe | PI209790 | Germany |
| 398 | Europe | PI531407 | Germany |
| 399 | Europe | PI232929 | Hungary |
| 400 | Europe | PI289320 | Hungary |
| 401 | Europe | PI289321 | Hungary |
| 402 | Europe | PI289324 | Hungary |
| 403 | Europe | PI290726 | England |
| 404 | Europe | PI346934 | Ukraine |
| 405 | Europe | PI346941 | Ukraine |
| 406 | Europe | PI346942 | Ukraine |
| 407 | Europe | PI346933 | Soviet Union |
| 408 | Europe | PI346935 | Soviet Union |
| 409 | Europe | PI346944 | Soviet Union |
| 410 | Europe | PI346945 | Soviet Union |
| 411 | Europe | PI442533 | Belgium |
| 412 | Europe | PI516181 | Romania |
| 413 | Europe | PI531399 | Bulgaria |
| 414 | Europe | PI531402 | Czechoslovakia |
| 415 | Europe | PI531406 | Czechoslovakia |
| 416 | 12 South America | PI202294 | Argentina |
| 417 | South America | PI202295 | Argentina |
| 418 | 13 North America | PI296376 | Canada |
| 419 | North America | PI677103 | America |
| 420 | North America | PI649385 | America |
| 421 | North America | PI649384 | America |
| 422 | North America | PI649383 | America |
| 423 | 14 Oceania | PI365040 | Australia |
| 424 | Oceania | PI365842 | Australia |
| 425 | Oceania | PI365845 | Australia |
| 426 | Oceania | PI367683 | Australia |
| 427 | Oceania | PI367684 | Australia |
| 428 | 15 Africa | PI517016 | Morocco |
| 429 | Africa | PI517017 | Morocco |
| 430 | Africa | PI531419 | Kenya |
